# The Promoting Effect of the Extracellular Matrix Peptide TNIIIA2 Derived from Tenascin-C in Colon Cancer Cell Infiltration

**DOI:** 10.3390/ijms18010181

**Published:** 2017-01-17

**Authors:** Hideo Suzuki, Manabu Sasada, Sadahiro Kamiya, Yuka Ito, Hikaru Watanabe, Yuko Okada, Kazuma Ishibashi, Takuya Iyoda, Akinori Yanaka, Fumio Fukai

**Affiliations:** 1 Department of Gastroenterology, Institute of Clinical Medicine, University of Tsukuba Graduate School, 1-1-1 Tennodai, Tsukuba, Ibaraki 305-8575, Japan; 2 Faculty of Pharmaceutical Sciences, Tokyo University of Science, 2641 Yamazaki, Noda, Chiba 278-8510, Japan; ssdy1321.tus@gmail.com (M.S.); yuka02079@gmail.com (Y.I.); hika.w.12v345key@gmail.com (H.W.); bitawan850@gmail.com (Y.O.); k.ishibashi0604@gmail.com (K.I.); iyoda@rs.tus.ac.jp (T.I.); fukai@rs.noda.tus.ac.jp (F.F.); 3 Department of Drug Informatics, Faculty of Pharmaceutical Sciences, Josai International University, 1 Gumyo, Togane, Chiba 283-8555, Japan; kamiyas@jiu.ac.jp; 4 Department of Gastroenterology, Hitachi Medical Education and Research Center, Tsukuba University Hospital, 2-1-1 Jyounancho, Hitachi, Ibaraki 317-0077, Japan; ynk-aki@md.tsukuba.ac.jp

**Keywords:** extracellular matrix, tenascin C, TNIIIA2, matrix metalloproteinase, colon cancer

## Abstract

The extracellular matrix (ECM) molecule tenascin C (TNC) is known to be highly expressed under various pathological conditions such as inflammation and cancer. It has been reported that the expression of TNC is correlated with the malignant potential of cancer. In our laboratory, it was found that the peptide derived from the alternative splicing domain A2 in TNC, termed TNIIIA2, has been shown to influence a variety of cellular processes, such as survival, proliferation, migration, and differentiation. In this study, we investigated the effect of TNC/TNIIIA2 on the invasion and metastasis of colon cancer cells, Colon26-M3.1, or PMF-Ko14, using an in vitro and in vivo experimental system. The degree of cell invasion was increased by the addition of TNC and TNIIIA2 in a dose-dependent manner. The invasion by TNC and TNIIIA2 were suppressed by an MMP inhibitor or TNIIIA2-blocking antibody. In an in vivo experiment, pulmonary metastasis was promoted conspicuously by the addition of TNIIIA2. In this study, we found that colon cancer cell invasion and metastasis was accelerated by TNC/TNIIIA2 via MMP induction. This result suggests the possibility of a new strategy targeting TNC/TNIIIA2 for colon cancer.

## 1. Introduction

Many internal cells adhere and depend on the extracellular matrix (ECM). The ECM participates in various processes, including cell proliferation, survival/death, differentiation, migration, and adhesion [1,2]. This “anchorage dependent” cell functions are controlled mainly by integrin, a transmembrane adhesion receptor. Integrin is expressed on the cell membrane as a heterodimer of two subunits, called the α chain and β chain. Until now, 19 types of α chains and eight types of β chains have been found, and at least 24 types of different integrin heterodimers were identified by those combinations [3]. When cells are combined with ECM through integrin, the cytoskeleton system is constructed in the intracellular domain of integrin and activates various signal molecules [4].

Tenascin C (TNC), one of the ECM components, forms a large structure body outside of the cell by assembling other ECM and participates in cell adhesion, movement, permeation, survival/migration, and differentiation [5]. Depending on the cellular context, TNC acts as either an adhesive or an antiadhesive substrate [6,7,8]. Because TNC has contradicting actions in cell adhesion, it is classified as adhesion modulatory ECM. TNC is not expressed in normal cells except in immune tissues, such as bone marrow and thymus gland [9,10], but is expressed specifically in malignancy, inflammation, and wound healing. It is reported that the high expression of a molecular variant of TNC was elevated depending on a malignant potential in the stroma of some malignancies, including oral cancer, sarcoma, breast cancer, and colon cancer [11,12,13,14]. TNC is a large protein with a molecular weight of 250–350 kDa, which is composed a hexamer connecting the polypeptide chain at the N-terminus and a peptide derived from the selective splice domain A2, called TNIIIA2 [15]. TNIIIA2 is a 22-mer peptide containing an active sequence YTITIRGV (human), and it has various influences on cell function as well as the promotion and induction of cell adhesion by the strong and continuous activation of β1 integrin through the syndecan-4 receptor [16], but the expression level of TNIIIA2 in vivo has not been well known.

However, the morbidity and mortality of colon cancer have recently increased in Japan, ranking as the third and second most common causes of morbidity and the third and first most common causes of mortality in males and females, respectively. Risk factors for colon cancer include environmental factors such as overeating, drinking, and processed meat [17] as well as hereditary diseases such as familial adenomatous polyposis [18,19]. The increased expression of TNC has been reported in both inflammatory bowel disease patients and colon cancer patients [14,20,21]. In addition, there is an association between malignant potential and TNC expression for colon cancer [14], and it is possible that TNC participates in the malignant transformation of tumors. The increased expression of TNC macromolecule variant that develops in malignant tumor tissue participates in tumor formation, migration, and malignant transformation [22]; however, the detailed mechanism by which TNC participates in such a pathological process based on the sequences involved in biochemical activity is not clear. Therefore, we performed the following analysis to clarify the participation of TNC and the functionality of the peptide TNIIIA2 isolated by TNC in the invasion and metastasis of colon cancer cells in this study.

## 2. Results

### 2.1. Functional Analysis of Tenascin C (TNC)/TNIIIA2 in the Invasion of Colon Cancer Cells

**Figure 1:**
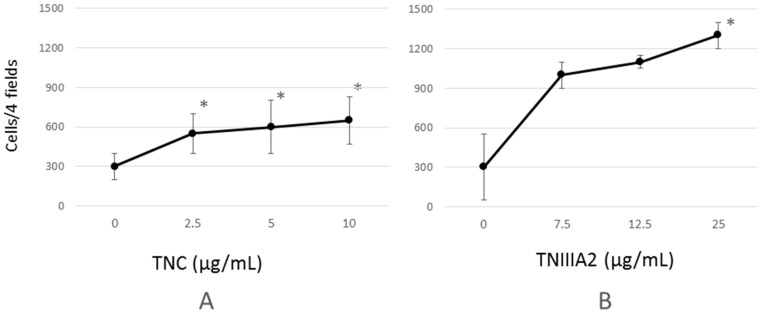
Tenascin C (TNC) and the peptide derived from the alternative splicing domain A2 in TNC (TNIIIA2) dose-dependently promote Colon26-M3.1 invasion. Colon26-M3.1 cells (3.0 × 10^4^ cells/200 μL/well) were seeded into the upper compartment of the invasion chamber in the presence of TNC (**A**) or TNIIIA2 (**B**) and were allowed to invade for 24 h. Cells that moved into the lower surface of the membranes were stained with crystal violet and counted for four high powered fields. Data represent the mean of three determinations ± SD. * *p* < 0.05.

An invasion assay was performed to clarify whether TNC or TNIIIA2 had an influence on the sub-basal membrane invasion of Colon26-M3.1 cells. As a result, both TNC and TNIIIA2 increased invasion in a concentration-dependent manner, and their invasion reached plateau (Figure 1). Additionally, the results showed that TNC induced Colon26-M3.1 cell invasion significantly but only weakly, while TNIIIA2 potently induced invasion, but required higher concentrations. 

**Figure 2:**
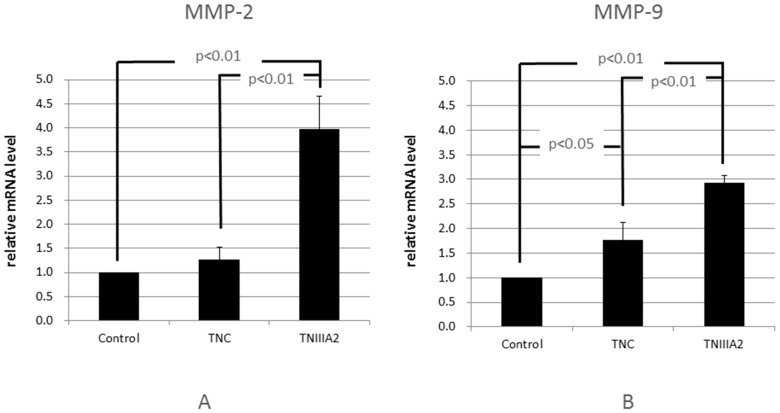
TNC and TNIIIA2 upregulate the mRNA level of matrix metalloproteinase (MMP)-2,9 of Colon26-M3.1. Colon26-M3.1 cells (2.5 × 10^5^ cells/500 μL/well) were seeded onto a 24-well plate in the presence or absence of TNC (10 µg/mL) or TNIIIA2 (25 µg/mL) in serum-free medium. After incubation for 48 h at 37 °C, the mRNA levels of MMP-2 (**A**) and MMP-9 (**B**) were quantified by real-time RT PCR. Data represent the mean of three determinations ± SD.

Matrix metalloproteinase (MMP) is an enzyme that breaks down ECM. In the invasion of cancer cells to the sub-basal membrane, MMP produced by cancer cells destroys the basal membrane enzymatically and the cancer cells move into that gap [23,24]. It is known that both TNC and MMP are strongly expressed in tumor tissue [25]; in addition, there are reports that TNC aggravates the MMP production of cancer cells [26,27]. Therefore, we hypothesized that TNC or TNIIIA2 affected the promotion of MMP and examined the mRNA expression of MMP-2/9 of Colon26-M3.1 via RT-PCR. The results showed that TNC was hardly induced in *MMP-2* gene expression, but significantly, albeit weakly, in *MMP-9* gene expression. In contrast, TNIIIA2 prominently increased the expression of both *MMP-2* and *MMP-9* gene (Figure 2). The results suggest that TNC might have an ability, albeit a weak one, to induce colon cell invasion at lower concentrations, while TNIIIA2 potently induce the invasion but requires higher concentrations, as compared to that of TNC.

**Figure 3:**
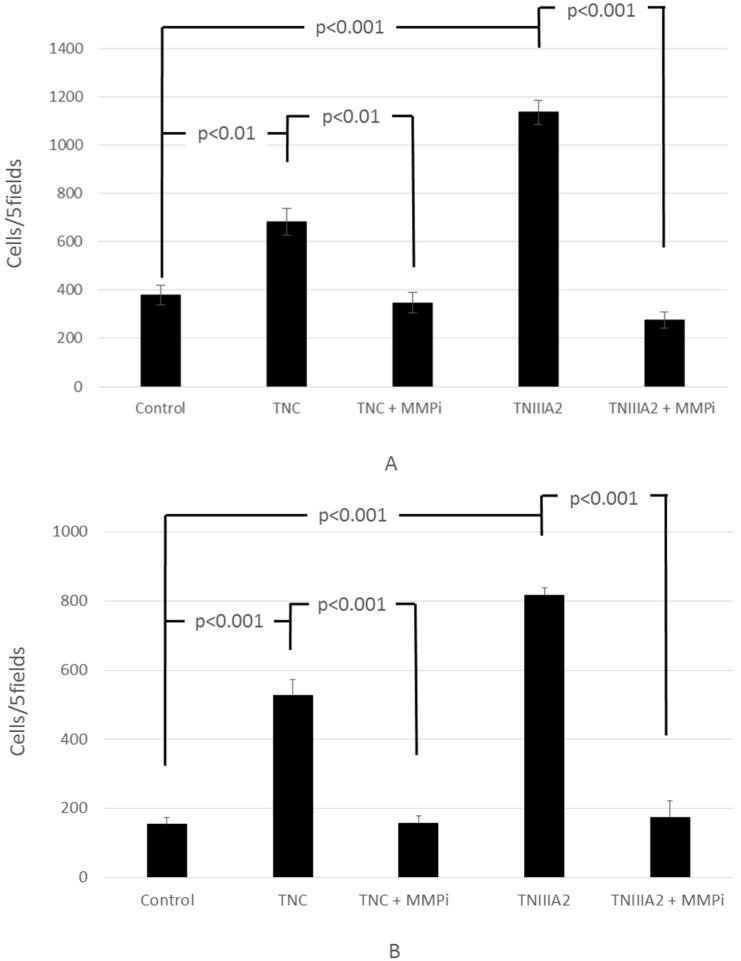
The enhanced invasion of Colon26-M3.1 (**A**) or PMF-Ko14 (**B**) by TNC and TNIIIA2 is dependent on Matrix metalloproteinase (MMP). Colon26M3.1 cells (3.0 × 10^4^ cells/200 μL/well) or PMF-Ko14 cells (3.0 × 10^4^ cells/100 μL/well) were seeded onto the upper compartment of the invasion chamber with TNC (10 µg/mL) or TNIIIA2 (12.5 µg/mL) and an MMP inhibitor (100 µM) and were allowed to invade for 24 h. Cells that invaded the lower surface of the membranes were counted. Data represent the mean of three determinations ± SD.

Next, we analyzed the action of TNC on invasion in the presence of MMP inhibitor. The invasion by TNC and TNIIIA2 was completely blocked by an MMP inhibitor in Colon26-M3.1 (Figure 3A) and PMF-Ko14 (Figure 3B), suggesting that MMP-2/9 secretion from these cells was involved in their invasion induced by either TNC or TNIIIA2.

**Figure 4:**
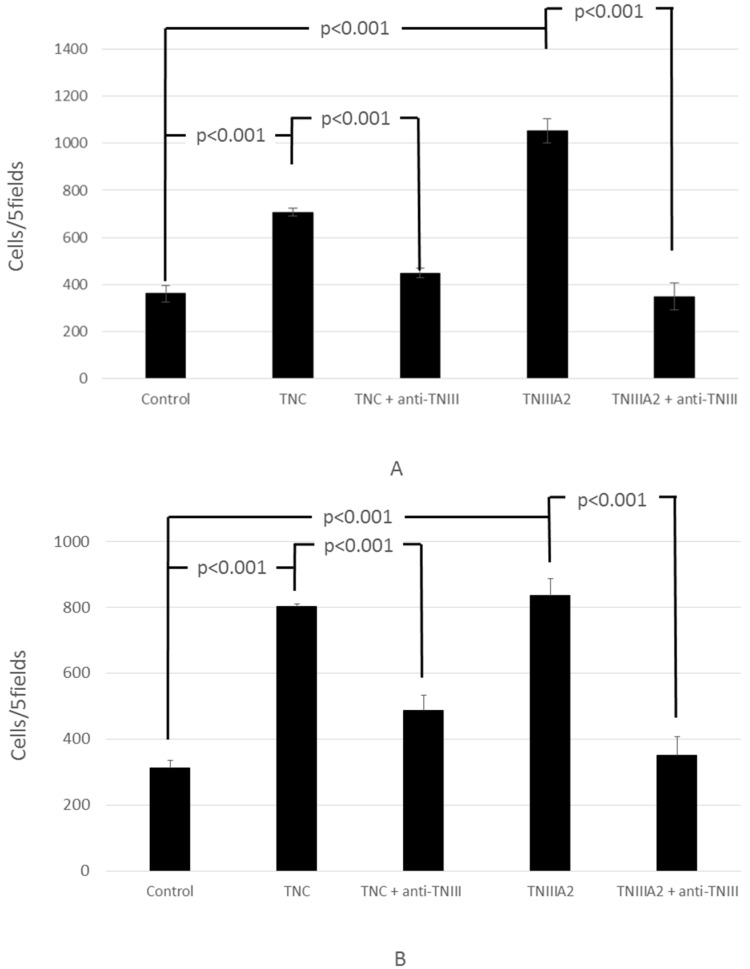
Involvement of anti-TNIIIA2 inhibitor in the increase of Colon26-M3.1 (**A**) or PMF-Ko14 (**B**) invasion by TNC and TNIIIA2. Colon26-M3.1 cells (3.0 × 10^4^ cells/200 μL/well) or PMF-Ko14 cells (3.0 × 10^4^ cells/100 μL/well) were seeded onto the upper compartment of the invasion chamber with TNC (10 µg/mL) or TNIIIA2 (12.5 µg/mL) and an anti-TNIIIA2 antibody (10 µg/mL) and were allowed to invade for 24 h. Cells that invaded the lower surface of the membranes were stained with crystal violet and counted for four high-powered fields. Data represent the mean of three determinations ± SD.

We previously reported that MMP-2/9 promotes the limited proteolysis of TNC itself as well as the basal membrane ECM and isolates the functional component with TNIIIA2 activity [16]. The invasion-promoting effect of Colon26-M3.1 and PMF-Ko14 by TNC mentioned above might be a component of the effects of isolated TNIIIA2-associated fragments, which were promoted by MMP. Therefore, we performed invasion experiments by adding an anti-TNIIIA2 antibody. The results show that the invasion-promoting action of TNC and TNIIIA2 was inhibited by the anti-TNIIIA2 antibody (Figure 4). 

These results suggest that at least one part of the invasion-promoting action of TNC is related to the TNIIIA2 component in TNC molecules. In other words, MMP production is promoted by TNC, and the mechanism that exposes TNIIIA2 by degradation promotes further invasion. Taken together with the results of Figure 1, Figure 2 and Figure 3, it could be assumed that MMP-9 secretion induced by TNC leads TNIIIA2 release from TNC, and the resulting TNIIIA2 further induces induction of *MMP-2/9* gene, resulting in active invasion of Colon26-M3.1 cells and PMF-Ko14 cells. Thus, weak secretion of MMP-9 induced by TNC may trigger a positive spiral of *MMP-2/9* gene expression through release of TNIIIA2.

### 2.2. The Effect of TNIIIA2 on a Mouse Model of Metastasis

**Figure 5:**
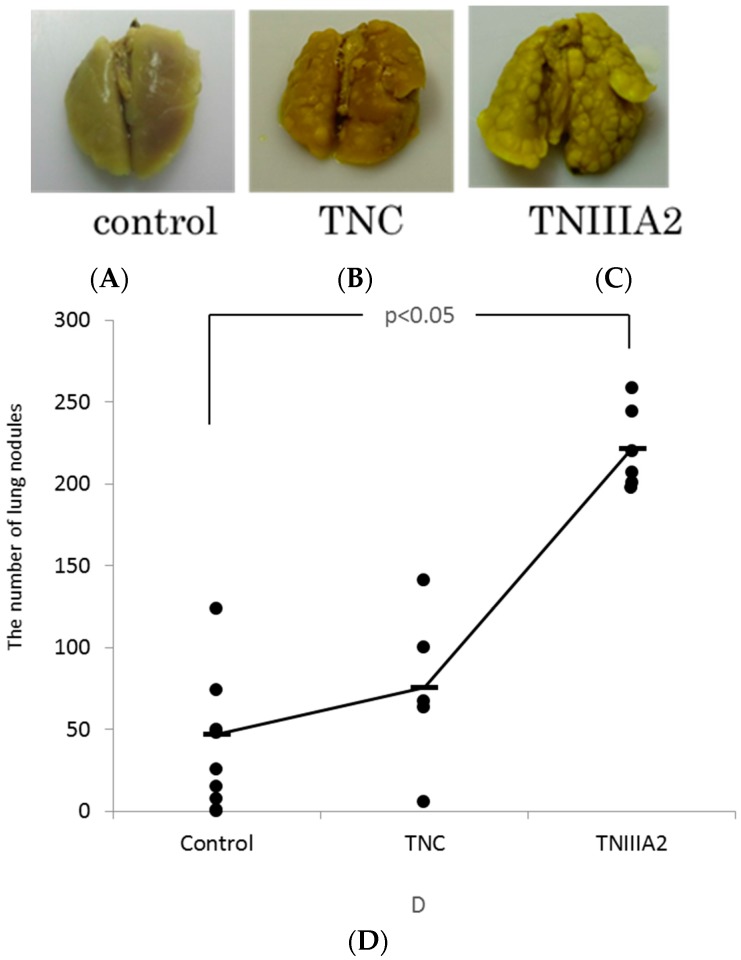
TNIIIA2 increases the number of lung metastatic nodules. A Colon26-M3.1 cell suspension (5.0 × 10^4^ cells/100 μL/head) was injected intravenously into six-week-old Balb/c female mice with or without TNC (5 μg/head) or TNIIIA2 (5 µg/head, *n* = 5 each). Fourteen days after injection, the lungs were stained with Bouin’s fixative. (**A**) The overview of the lungs of mice injected with Colon26-M3.1 alone, (**B**) those with TNC, and (**C**) those with TNIIIA2. The number of lung metastatic nodules was counted (**D**).

The results from in vitro experiments suggest that TNC and TNIIIA2 induce cancer cell invasion with producing MMP and might be related to the cell growth factor of mesenchymal cells and the induction of cytokine production. Next, we analyzed the action of TNC and TNIIIA2 using a pulmonary metastasis model by injecting Colon26-M3.1 cells into the mouse tail vein. The results show that the number of the pulmonary nodules increased clearly by the addition of TNC and TNIIIA2. The addition of TNIIIA2 showed a statistically significant increase in nodules compared to controls (Figure 5).

## 3. Discussion

TNC is a multifunctional ECM with a variety of actions, and it may have a relationship with the malignant potential of colon cancer [14], but its functions are not well understood. 

In this study, it was revealed that the production of MMP-2/9 was increased by TNC or TNIIIA2. We previously reported that the proteolysis of TNC by MMP induced the release of functional TNC fragments, including TNIIIA2, resulting in the activation of β1 integrin [16]. In addition, Notch signaling plays important roles in carcinogenesis in colon cancer [28]. Because Notch signaling is thought to be related to MMP-9 activation, TNC or TNIIIA2 might activate Notch signals through the production of MMP and participate in tumor invasion. The invasion-promoting effect of TNIIIA2 is presumed to participate in the process by which the functional component of TNIIIA2 is exposed by limited proteolysis via MMP production and the activation of β1 integrin accelerates further invasion.

In in vivo experiments that evaluated the pulmonary metastasis of colon cancer cells, pulmonary metastasis was facilitated by the combination of TNIIIA2. Two types of metastasis are prevalent: one is hematogenous metastasis, in which cancer cells detach from the primary tumor and spread through the blood to other organs, and the other is lymphogenous metastasis, in which the cancer cells spread through the lymph. In this experiment, we injected cancer cells into the tail vein, which imitated hematogenous metastasis. Hematogenous metastasis is thought to be constructed in several steps. The steps include detachment from a primary tumor, permeation or invasion into the blood vessels, migration from intravascular to extravascular regions, and adhesion or engraftment to organs such as the lungs or the liver. In this study, it was revealed that TNIIIA2 participates in hematogenous metastasis after cancer cells infiltrate the blood vessels. Oskarsson et al. reported that TNC derived from cancer cells contributed to pulmonary micrometastasis and played important roles in the initial stage of tumor growth [29]. It is thought that the difference in the survival/proliferation of TNIIIA2 depends on the cell strain. Further detailed analysis, including the investigation of human cells in which TNC is highly expressed or TNIIIA2 is involved in metastasis, is required.

Although it is not clear how TNC or TNIIIA2 participate in cancer invasion, fibroblastic cells have been reported to be involved in the process. The tumor stroma is composed of fibroblastic cells, inflammatory cells such as macrophages and various factors, including the ECM protein. The interaction between epithelial cells and stroma cells plays an important role in the carcinogenic process, including hyperplasia from normal epithelial cells, the formation of adenoma and malignant transformation [30]. The TGF-β released from fibroblastic cells is reported to affect the malignant transformation of cancer cells [31] and promote the function of TNC and participation in cancer invasion [32]. The relationship between fibroblastic cells and TNIIIA2 has not been clarified, and future investigation is necessary.

Some reports have mentioned the possibility that the anticancer agent targets TNC. Santis et al. reported that the monoclonal antibody ST2146 for TNC showed good accumulation in tumor tissue regardless of the expression of TNC by the analysis of tumor-bearing mice [33]. They suggested that the chimeric antibody of TNC, which showed high accumulation in brain tumors, could be used as a therapeutic drug [34]. Other clinical trials have been carried out with some TNC-specific antibodies [35]. In addition, because the serum and immunohistochemical TNC level is high in colon cancer and bladder cancer patients, it can act as a tumor marker [36,37,38]. TNIIIA2, which we analyzed in this study, might be useful as a tumor marker as well as a target of anticancer agents. 

This study has some limitations. Most importantly, we are not able to declare that the data obtained in this study could be applied widely in human malignancies other than colon cancer. Other cancers should be examined in addition to colon cancer. It would be important to ascertain if the anti-cancer effect of anti-TNIIIA2 antibody could be successfully observed also in other cancer cells and another in vivo xenograft model.

## 4. Materials and Methods

### 4.1. Cells and Animals

The mouse colon cancer cell line Colon26-M3.1 was acquired from Saiki at the Division of Pathogenic Biochemistry, Institute of Natural Medicine, University of Toyama, Toyama, Japan. Colon26-M3.1 is a mouse epithelial-like transformed colon cell line and well known to induce lung metastasis by vein injection. Roswell Park Memorial Institute RPMI1640 medium (Nissui Pharmaceutical Co., Ltd., Tokyo, Japan) with 10% FBS (SAFC Biosciences, St. Louis, MO, USA) with 0.15% NaHCO_3_, 2 mM l-glutamine, and 100 µg/mL penicillin-streptomycin (Nacalai Tesque, Kyoto, Japan) was used for Colon26-M3.1 cell culture. The PMF-ko14 cell was obtained from RIKEN Bio Resource Center (Tsukuba, Japan). This cell originated from a Japanese colon cancer patient and was established as cell line. Dulbecco’s Modified Eagle’s Medium (DMEM) (Sigma-Aldrich Japan Co., Ltd., Tokyo, Japan) with 10% FBS (SAFC Biosciences, St. Louis, MO, USA) was used for PMF-ko14 cell culture. Cells were incubated in a 5% CO_2_ incubator at 37 °C.

The Balb/c mice were purchased from Sankyo Laboratory Service Co., Inc. (Tokyo, Japan).

### 4.2. Reagents and Instruments

TNC was purified from the culture supernatant of the SK-MEL-28 cells according to methods reported elsewhere [39]. Refined TNC was confirmed to include a high polymer variant of molecular weight 250–350 kDa. The cell counting kit and Engelbreth–Holm–Swarm (EHS) sarcoma extract (Matrigel^®^) were purchased from Dojindo (Kumamoto, Japan) and AGC Asahi Glass (Tokyo, Japan), respectively. The synthetic TNIIIA2 peptide (RSTDLPGLKAATHYTITIRGVTC) was purchased from Eurofins genomics (Whitefield, India). The preparation of antihuman TNIIIA2 polyclonal antibody has been described previously [40]. In brief, the synthetic peptide CATHYTITIRGVT was conjugated to KLH (keyhole limpet hemocyanin) as a haptenic antigen and it was immunized to a rabbit. The IgG fraction of rabbit serum was applied to Sepharose beads coupled with the synthetic peptide immunogen. Eluted IgG was used as the anti-TNIIIA2 antibody. The MMP-2/-9 inhibitor II, (2*R*)-((4-biphenylylsulfonyl)-amino)-*N*-hydroxy-3-phenylpropionamide (BiPS) was obtained from Calbiochem (San Diego, CA, USA). The QuantiFast SYBR Green PCR Kit was purchased from QIAGEN (Hilden, Germany). The Nuclepore Track-Etch Membrane was obtained from Sigma-Aldrich (St. Louis, MO, USA). We used HITACH MTP-800 Lab (Tokyo, Japan) for absorbance measurements and the Applied Biosystems 7500 Real-Time PCR system (San Francisco, CA, USA) for RT-PCR.

### 4.3. Invasion Assay

A transwell chamber was used for invasion assays [41]. The upper chamber and lower chamber were sterilized by UV irradiation. The porous PVP membrane filter (8.0 µm) was sterilized by autoclave. Two hundred microliters of medium including 5% FBS was added to the lower chamber and the membrane filter mentioned above was placed on top without creating air bubbles; this assembly was attached to the upper chamber. For a basement membrane matrix, EHS sarcoma extract (Matrigel^®^) including 1 µg/mL FN was adjusted to 600 µg/mL with PBS solution, and 100 µL were applied to the membrane filter. The Matrigel^®^ was gelated by incubation at 37 °C in a 5% CO_2_ incubator for one hour. After removing the supernatant of the gel, Colon26-M3.1 cells (3.0 × 10^4^ cells/200 µL/well) or PMF-ko14 cells (3.0 × 10^4^ cells/100 μL/well) were seeded and incubated at 37 °C in a 5% CO_2_ incubator for 24 h with various concentrations of TNC or TNIIIA2 with or without an MMP inhibitor or an anti-TNIIIA2 antibody. After removing non-permeating cells from the membrane filter with a cotton swab, the invaded cells were fixed by dipping them in a PBS (−) solution including 4% formalin and 10% glycerol with the undersurface as the top at room temperature for one hour. The invaded cells were counted in 4–5 areas under a 100× microscope after dying the cells with crystal violet stain.

### 4.4. RT-PCR

Colon26M3.1 cells (2.5 × 10^5^ cells/500 μL/well) were seeded onto a 24-well plate in the presence or absence of TNC (10 µg/mL) or TNIIIA2 (25 µg/mL) in a serum-free medium. The cell RNA was extracted using the PCR RNeasy^®^ Mini Kit (QIAGEN, Hilden, Germany) according to the manufacturer’s instructions. After the RNA concentration was measured by Nano Drop (ND-1000 spectrophotometer), cDNA was gathered by the reverse transcription reaction using Quanti Tect^®^ Reverse Transcription (QIAGEN, Hilden, Germany) and amplified in TaKaRa PCR Thermal Cycler Dicer (Takara Bio, Shiga, Japan) using various primers (Table 1).

The PCR products were electrophoresed using 2% agarose gel TBE including 0.5 µg/mL EtBr and developed with a trans-illuminator.

### 4.5. Animal Model of Metastasis

The Colon26-M3.1 cells suspended in medium including 10% FBS with or without TNC (5 µg/head) or TNIIIA2 (5 µg/head) were injected to the tail vein of the female Balb/c mice (6 weeks old, *n* = 5 each). The lungs were removed 14 days after injection and fixed and stained with Bouin’s solution. Metastatic foci can be distinguished as whitish colonies from yellowish lung parenchyma. Surface metastatic foci in lung lobes were counted under a dissecting microscope. All of the animal procedures were approved by the Institutional Animal Care and Use Committee (IACUC) of Tokyo University of Science (protocol # Y12032, Y13037, Y14039, Y15002, Y16052; date of approval is 28 March 2012).

### 4.6. Statistical Analysis

All values are shown as the means ± standard deviation. A two-tailed Student’s *t* test was used to analyze the difference between the two groups, and the Student–Newman–Keuls post-hoc test was used for more than three groups. Statistical analyses were performed with the software Statcel 4 (OMS Publishing Co., Saitama, Japan) and SAS 9.4 (SAS Institute, Cary, NC, USA). A statistically significant difference was represented by a *p*-value less than 0.05.

## 5. Conclusions

In this study, we showed that TNC participated in the malignant transformation of colon cancer cells through one of its functional components, TNIIIA2. Thus, a new therapeutic strategy for colon cancer could target TNC/TNIIIA2, and further investigation is needed.

## Figures and Tables

**Table 1:** The primers for MMP-2 and MMP-9.

GAPDH	Forward: 5′-TTCACCACCATGGAGAAGGC-3′
	Reverse: 5′-GGCATGGACTGTGGTCATGA-3′
MMP-2	Forward: 5′-AGATCTTCTTCTTCAAGGACCGGTT-3′
	Reverse: 5′-GGCTGGTCAGTGGCTTGGGGTA-3′
MMP-9	Forward: 5′-CACCACCACAACTGAACC-3′
	Reverse: 5′-GCCTAGACCCAACTTATCC-3′
